# Involvement of the nuclear factor-κB signaling pathway in the regulation of CXC chemokine receptor-4 expression in neuroblastoma cells induced by tumor necrosis factor-α

**DOI:** 10.3892/ijmm.2014.2032

**Published:** 2014-12-10

**Authors:** YUNLAI ZHI, HONGTING LU, YUHE DUAN, WEISHENG SUN, GE GUAN, QIAN DONG, CHUANMIN YANG

**Affiliations:** 1 Department of Pediatric Surgery, The Affiliated Hospital of Qingdao University, Qingdao, Shandong 266003, P.R. China; 2 Department of Pediatric Surgery, The Children’s Hospital of Zhengzhou, Henan 450053, P.R. China; 3 Department of Organ Transplantation Center, The Affiliated Hospital of Qingdao University, Qingdao 266003, P.R. China

**Keywords:** tumor necrosis factor-α, nuclear factor-κB, CXC chemokine receptor-4, neuroblastoma, metastasis

## Abstract

Metastasis is a hallmark of malignant neuroblastoma and is the main reason for therapeutic failure and recurrence of the tumor. The CXC chemokine receptor-4 (CXCR4), a Gi protein-coupled receptor for the ligand CXCL12/stromal cell-derived factor-1α (SDF-1α), is expressed in various types of tumor. This receptor mediates the homing of tumor cells to specific organs that express the ligand, CXCL12, for this receptor and plays an important role in tumor growth, invasion, metastasis and angiogenesis. In the present study, the inflammatory cytokine, tumor necrosis factor-α (TNF-α) upregulated CXCR4 expression in neuroblastoma cells and increased migration to the CXCR4 ligand SDF-1α. In addition, this effect was dependent upon NF-κB transcriptional activity, as blocking the NF-κB pathway with pyrrolidinedithiocarbamic acid ammonium salt suppressed TNF-α-induced upregulation of CXCR4 expression and reduced the migration towards the CXCR4 ligand, SDF-1α. Treating neuroblastoma cells with TNF-α resulted in the activation of nuclear factor-kappa B (NF-κB) and subsequently, the translocation of NF-κB from the cytoplasm to the nucleus. Using immunohistochemistry, NF-κB and CXCR4 were significantly correlated with each other (P=0.0052, Fisher’s exact test) in a cohort of neuroblastoma samples (n=80). The present study indicates that the inflammatory cytokine, TNF-α, partially functions through the NF-κB signaling pathway to upregulate CXCR4 expression to foster neuroblastoma cell metastasis. These findings indicate that effective inhibition of neuroblastoma metastasis should be directed against the inflammatory cytokine-induced NF-κB/CXCR4/SDF-1α signaling pathway.

## Instructions

Neuroblastoma, the most common extracranial solid tumor in children, is a heterogeneous tumor that arises from the neural crest (1). Patients with neuroblastoma account for ~15% of childhood fatalities from cancer. At the time of diagnosis, >70% of patients have metastatic disease (2,3). The disease displays a remarkable clinical diversity, ranging from spontaneous regression to fatal progression and dissemination to privileged sites, such as bone-marrow and liver (4,5). However, the molecular mechanisms and/or intrinsic factors controlling neuroblastoma cancer metastasis are not well understood.

The tumor microenvironment plays a crucial role in orchestrating immune cell effectors/modulators, pro- and anti-inflammatory cytokines, and chemokine production. The tumor microenvironment does this by impacting, integrating and subverting the immunity and inflammatory processes (6–9). Numerous studies have demonstrated that the tumor microenvironment not only responds to and supports carcinogenesis, but also actively contributes to tumor initiation, progression and metastasis (10). Mediators of inflammation have long been known to increase metastatic dissemination (11,12). Furthermore, inflammatory cytokines in the tumor microenvironment promote nuclear factor-κB (NF-κB) signaling pathways activation, which may induce the expression of several genes associated with malignant transformation (13).

Previously, it has become clear that NF-κB signaling also has a critical role in cancer development and progression (14). NF-κB actions as a dimer composed of the RelA (p65) and NF-κB1 (p50) or NF-κB2 (p52) subunits. In normal resting cells, NF-κB is sequestered in the cytoplasm through binding to IκB. NF-κB activation involves its release from its inhibitor, IκB, and its subsequent translocation from the cytoplasm to the nucleus, where it binds to cognate sequences in the promoter region of multiple genes. Regulating gene expression by NF-κB is controlled mainly by the inhibitory IκB proteins, which include IκB-α. Upon stimulation, IκB-α is rapidly phosphorylated and degraded via the ubiquitin-proteasome pathway, permitting activation and nuclear import of NF-κB (15). NF-κB was also shown to induce the expression of CXCR4 (16).

Chemokines and their receptors were originally described as essential mediators of leukocyte directional migration, and have further emerged as crucial elements in all the stages of tumor development (17–19). The binding of chemokines to their cognate receptors elicits typical cellular responses, such as directional migration. CXCR4 is the most frequently expressed chemokine receptor on the tumor cells (20). The CXCR4 ligand is the small chemokine SDF-1α. In addition to its critical role in tumor cell growth, survival and angiogenesis in multiple cancers, the CXCR4/SDF-1α pair has been shown to mediate homing and metastatic secondary growth in SDF-1α-producing organs, such as liver and bone marrow (21–23) and a study indicates that neuroblastoma cells are equipped with a bone marrow homing system that may mediate the establishment of bone marrow metastasis by neuroblastoma cells (24).

To elaborate the role of the tumor microenvironment, particularly the inflammatory cytokines in neuroblastoma cells pathobiology, the NF-κB signaling activation was examined as a potential mechanism by which cell metastasis is fostered. In the present study, the expression of CXCR4 and NF-κB was examined in a subset of neuroblastoma tumors and correlative analyses were conducted with clinicopathological variables. Furthermore, the inflammatory cytokine TNF-α acts as a regulator of functional CXCR4 expression on neuroblastoma cells in a NF-κB-dependent manner. The inhibitor of NF-κB reduced CXCR4 expression on neuroblastoma cells and resulted in decreased migriation towards SDF-1α in response to TNF-α. Finally, the interaction of the tumor cells with macrophages was shown to enhance CXCR4 expression in the neuroblastoma cells in an NF-κB-dependent manner.

## Materials and methods

### Patients and tissue specimens

A total of 80 clinical neuroblastoma samples and 15 ganglioneuroma samples were collected from patients who had undergone surgical resection at the Department of Pediatric Surgery, The Affiliated Hospital of Qingdao University (Qingdao, Shandong, China) between January 2006 and November 2013. The patients who had undergone preoperative chemotherapy and radiotherapy were not included. Hematoxylin and eosin-stained slides from all the cases were reviewed to confirm the diagnoses. All the neuroblastoma samples were classified according to the International Neuroblastoma Staging System (INSS) classification criteria (25). The 80 patients included 48 males and 32 females aged from 1 month to 11 years (median, 5 years). The clinicopathological characteristics of the 80 patients are summarized in Table I. The patient consent was obtained, and the study was approved by the Institutional Ethics Review Committee of the Affiliated Hospital of Qingdao University.

### Immunohistochemistry (IHC)

Immunohistochemistry for RELA (P65) and CXCR4 was performed. All the specimens were fixed with 4% formaldehyde, paraffin-embedded and cut into 4-*μ*m serial sections. Following deparaffinization and rehydration, tissue slides were cooked to retrieve the antigen in a microwave oven with 10 mM citrate buffer (pH 6) for 15 min. Endogenous peroxidase activity was blocked with 3% hydrogen peroxide for 10 min at room temperature and washed with phosphate-buffered saline (PBS). Subsequently, the tissue slides were incubated with a purified rabbit polyclonal antibody against RELA (P65) (1:800, Cell Signaling Technology, USA) or CXCR4 (1:50, Abcam Cambridge, MA) at 4°C overnight. Following washing with PBS, the sections were incubated with secondary antibodies (goat anti-rabbit polyclonal antibody) for 30 min at 37°C. The sections were subsequently washed three times with PBS and treated with diaminobenzidine. Finally, the slides were counterstained with hematoxylin, dehydrated and mounted. Negative controls were probed with PBS under the same experimental conditions.

### Histological assessment

All the samples were evaluated in a blinded manner by two independent pathologists without knowledge of any other clinicopathological data. The score method to evaluate the immunostaining results was performed by multiplying the stain intensity by the stain area. The stain intensity score was as follows: No staining (score 0), weak staining (score 1), moderate staining (score 2) or strong staining (score 3). The percentage of the extent of reactivity was scored as follows: <25% (score 1), 25–50% (score 2), 50–75% (score 3) or >75% (score 4) of tumor cells. The total expression of NF-κB-p65 and CXCR4 was determined as either negative or low expression (−), score ≤2; or positive or high expression (+), score ≥3.

### Cell culture and reagents

The human malignant neuroblastoma SH-SY5Y cell lines and human monocytic cell line THP-1 were obtained from the Cell Bank of Type Culture Collection of Chinese Academy of Science (Shanghai, China). The cell lines were maintained in Dulbecco’s Modified Eagle’s Medium (DMEM; Hyclone, Logan, USA) supplemented with 10% fetal bovine serum (FBS; Hyclone), and the cells were cultured at 37°C with 5% CO_2_ in humidified atmosphere. Recombinant human TNF-α (Sigma-Aldrich, St. Louis, MO, USA) was dissolved in 0.1% bovine serum albumin (BSA) in PBS, and stock solution (10 *μ*g/ml) was stored at −20°C. Recombinant human SDF-1α (Life Technologies, Carlsbad, CA, USA) was prepared in 0.1% BSA in PBS and stock solution (100 *μ*g/ml) was stored at 4°C. Phorbol myristate acetate (PMA), a THP-1 cells inducer (Sigma-Aldrich), was prepared in PBS (320 nM) and maintained at 4°C until required. Pyrrolidinedithiocarbamic acid ammonium salt (PDTC), a NF-κB (p65) inhibitor (3) (Sigma-Aldrich) was prepared in PBS (50 *μ*M) and maintained at 4°C until required.

### Co-culture of macrophage and SH-SY5Y cell lines

THP-1 cells (1×10^6^ per well) were seeded into the upper insert of a 6-well transwell apparatus with 0.4 *μ*m pore size (Corning Costar, Rochester, NY, USA) and treated with 320 nM PMA for 24 h to generate macrophages. Following a thorough wash to remove all PMA, PMA-treated THP-1 macrophages were co-cultured with SH-SY5Y cells (in a 6-well plate; 1×10^6^ cells per well) without direct contact. In the co-culture system, SH-SY5Y cells were cultured with THP-1-differentiated macrophages for 24 h and harvested for use in subsequent experiments.

### Inhibition of the NF-κB signaling pathway in the co-culture system

A concentration of 50 *μ*M PDTC was added to the macrophage/cancer cell co-cultures. After incubation for 24 h, the SH-SY5Y cells were harvested for RNA and protein extraction, then reverse transcription-quantitative polymerase chain reaction (RT-qPCR) and western blots were performed for CXCR4 and NF-κB-p65 quantification. Co-culture of SH-SY5Y and TAM without PDTC was the positive control and culture of SH-SY5Y alone without PDTC was the negative control.

### RT-qPCR

Total RNA was isolated from the cell lines by Trizol (Takara, Dalian, China) according to the manufacturers’ instructions. cDNA was synthesized using the Takara Prime Script RT reagent kit (Takara) in a total volume of 10 *μ*l, containing 2 *μ*l 5× Prime Script buffer, 0.5 *μ*l Prime Script RT Enzyme mix l, 0.5 *μ*l Oligo dT Primer (50 *μ*M), 0.5 *μ*l Random 6 mers (100 *μ*M) and 6.5 *μ*l total RNA. The conditions for RT were: 37°C for 15 min and 85°C for 5 sec. RT-qPCR was performed using the LightCycler system together with the LightCycler DNA Master SYBR Green I kit (Takara). The total volume was 20 *μ*l, containing 10*μ*l SYBR Premix Ex Taq II (2×), 0.8 *μ*l PCR Forward Primer (10 *μ*M), 0.8 *μ*l PCR Reverse Primer (10 *μ*M), 2.0 *μ*l template (<100 ng) and 6.4 *μ*l dH_2_O. The conditions for PCR were: 1 cycle at 95°C for 30 sec, and subsequently 40 cycles at 95°C for 5 sec and 20 sec at 60°C. The reference gene, glyceraldehydes-3-phosphate dehydrogenase (*GAPDH*) was used as an internal control. Gene expression was quantified by the comparative CT method, normalizing CT values to *GAPDH* and calculating the relative expression values. Primer sequences were as follows: *CXCR4*, forward 5′-TGGCTGAAAAGGTGGTCTAG-3′, and reverse 5′-GATGCTGATCCCAATGTAGT-3′. The amplification fragment was 333 basepairs (bp). *GAPDH* was used as the internal control, and the primer sequence was forward, 5′-TCATGGGTGAACCATGAGAATG-3′, and reverse 5′-GGCATGGACTGTGGTCATGAG-3′. The amplification fragment was 146 bp.

### Western blot analysis

The cells were washed twice with cold PBS (pH 7.0), and lysed in radioimmunoprecipitation assay buffer [150 mM NaCl, 1% Nonidet P-40, 1% deoxycholate, 0.1% SDS and 10 mM Tris-HCl (pH 8.0)] supplemented with protease inhibitors. The protein concentration of each sample was assayed using the bicinchoninic acid method kit (Pierce, Rockford, IL, USA). Equal amounts of protein (50 *μ*g) were subjected to SDS-PAGE on a 10% gel. Subsequently, the protein was blotted onto a polyvinylidene fluoride membrane. After blocking with 5% skimmed milk in 20 mM TBS with 0.1% Tween for 1 h at room temperature with agitation, the proteins were incubated with the indicated primary antibodies at 4°C overnight followed by incubation in mouse anti-rabbit secondary antibody conjugated with horseradish peroxidase (1:6000; Santa Cruz Biotechnology, Inc., Santa Cruz, CA, USA) for 1 h. The proteins were detected using the Pierce ECL Western Blotting substrate (Santa Cruz Biotechnology, Inc.), with a 15 min exposure after washing the membrane for imaging with the LAS-3000 image analyzer (Life Science, Fujifilm Global, Shanghai, China). The primary antibodies employed included anti-β-actin (1:2000; Santa Cruz Biotechnology, Inc.), anti-NF-κB-p65 (1:1000; Abcam, Cambridge, MA, USA), anti-CXCR4 (1:2000; Abcam), anti-p-IκB-α (1:1000; Santa Cruz Biotechnology, Inc.) and anti-IκB-α (1:1000; Santa Cruz Biotechnology, Inc.).

### Transwell migration assay

The assays were performed using a transwell (Corning Costar) containing a polycarbonate membrane filter (8-*μ*m pore size) for 24-well plates according to the manufacturer’s instructions. SH-SY5Y cells (5×10^5^) were pretreated for 24 h with TNF-α (20 ng/ml), the cells that were not exposed to TNF-α were used as the controls. Subsequently, the pretreated cells were harvested and seeded into the upper surface of the filter with a volume of 200 *μ*l DMEM containing 2% FBS in the presence or absence of PDTC (50 *μ*M) and placed into the lower wells containing 500 *μ*l complete medium with or without SDF-1α (100 ng/ml) to induce cell migration. The migration transwell chambers were incubated for 8 h at 37°C. Following incubation, the transwell chambers were washed twice with PBS and the cells on the bottom surface of the membrane were fixed in 95% ethanol for 10 min at room temperature, stained with 0.1% crystal violet for 30 min and washed three times with PBS. The number of migration cells in ten randomly selected microscopic fields (magnification, ×200) per membrane was counted.

### Statistical analysis

Statistical analysis was performed using the SPSS software 17.0. The data are expressed as the mean ± standard deviation. The Student’s *t*-test and one-way analysis of variance test were used to compare data between the different groups. The association between NF-κB-p65, CXCR4 expression and clinicopathological parameters was analyzed using the χ^2^ test or the Fisher’s exact test. The possible association of NF-κB-p65 and CXCR4 immunoreactivity was assessed using the Fisher’s exact test. P<0.05 was considered to indicate a statistically significant difference.

## Results

### Expression of NF-kB-p65 and CXCR4 in neuroblastoma compared to ganglioneuroma tissues

**Figure 1:**
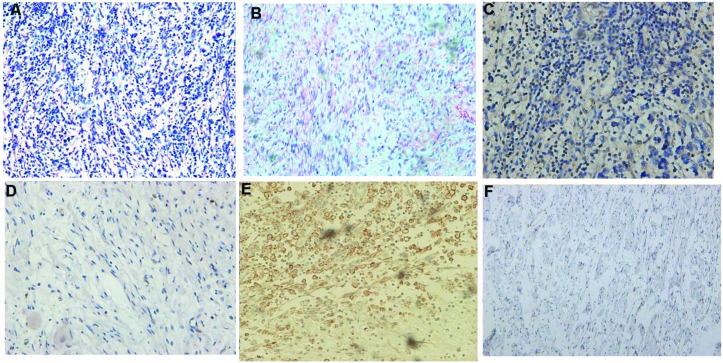
Immunohistochemical staining of NF-κB-p65 and CXCR4 in neuroblastoma and ganglioneuroma tissues. (A) Neuroblastoma tissues [hematoxylin and eosin (H&E) staining]. (B) Ganglioneuroma tissues (H&E staining). (C) High NF-κB-p65 expression in neuroblastoma tissues. (D) Low NF-κB-p65 expression in ganglioneuroma tissues. (E) High CXCR4 expression in neuroblastoma tissues. (F) Low CXCR4 expression in ganglioneuroma tissues. Original magnification, ×200. NF-κB, nuclear factor-κB; CXCR4, CXC chemokine receptor-4.

NF-kB-p65 protein showed cytoplasm and nucleus staining, while CXCR4 was observed predominantly at the cell membrane (Fig. 1). The expression rate of NF-kB-p65 positive in the neuroblastoma group was 73.6% (59/80), which was significantly higher than the rate of 20.0% (3/15) in the ganglioneuroma group, and the difference between the two groups was statistically significant (P=0.0001). The expression rate of CXCR4 positive was 70.0% (56/80) in the neuroblastoma group, which was significantly higher than the rate of 25.0% (3/12) in the ganglioneuroma group, and the difference between the two groups was statistically significant (P=0.0008) (Fig. 1, Table II).

### Expression of NF-kB-p65 and CXCR4 in neuroblastoma and their association with clinicopathological characteristics

**Figure 2:**
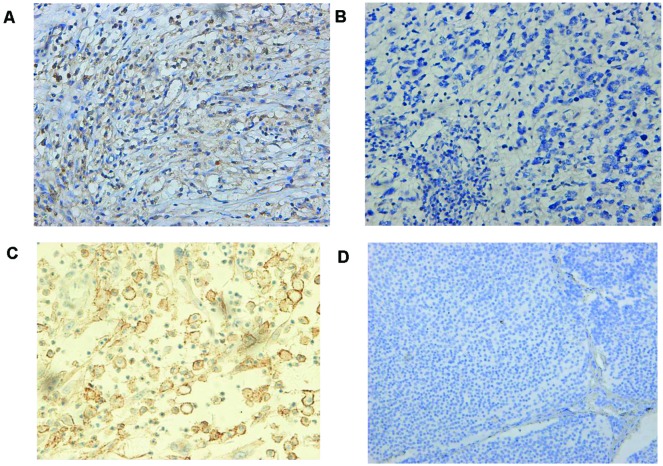
Immunohistochemical staining of NF-κB-p65 and CXCR4 in neuroblastoma samples. (A) High NF-κB-p65 expression in neuroblastoma tissues (INSS stage IV). (B) Low NF-κB-p65 expression in neuroblastoma tissues (INSS stage IIa). (C) High CXCR4 expression in neuroblastoma tissues (INSS stage IV). (D) Low CXCR4 expression in neuroblastoma tissues (INSS stage I). Original magnification, ×200. NF-κB, nuclear factor-κB; CXCR4, CXC chemokine receptor-4; INSS, International Neuroblastoma Staging System.

The level of NF-kB-p65 and CXCR4 expression was divided into the high and low groups according to the cut-off value stated in the aforementioned methods. There were significantly positive correlations between NF-kB-p65, CXCR4 expression and INSS stage (P=0.021) or metastasis (P=0.013). However, no statistical differences were found between clinicopathological factors (age, gender and tumor size) and NF-kB-p65, CXCR4 expression (Table I). Notably, there was a positive correlation between the expression of NF-kB-p65 and CXCR4 in the 80 neuroblastoma samples (P=0.0052, Fisher’s exact test) (Table III, Fig. 2).

### NF-kB contributes to TNF-α-mediated CXCR4 upregulation in neuroblastoma cells

**Figure 3:**
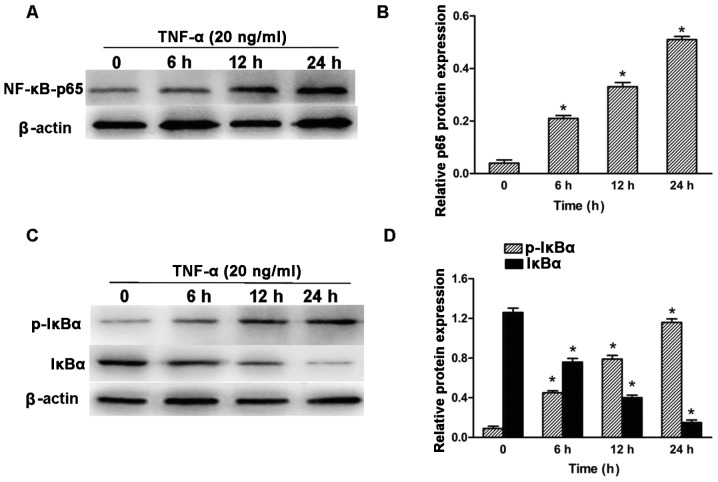
TNF-α-induces NF-κB activation and IκB-α phosphorylation in human SH-SY5Y cells. SH-SY5Y cells were treated with 20 ng/ml TNF-α for 0, 6, 12 and 24 h. (A and B) Cells were lysed following TNF-α treatment, and cell extracts were prepared and subjected to immunostaining for the p65 subunit of NF-κB or β-actin. (C and D) Whole cell lysates of TNF-α treated cells were immunoblotted for phosphorylated-IκB-α, total IκB-α and β-actin. β-actin was used as the loading control. Quantification of the target protein bands relative to β-actin is shown in the right panel. (^*^P<0.05 vs. untreated group). TNF-α, tumor necrosis factor-α; NF-κB, nuclear factor-κB.

As the levels of CXCR4 appeared to correlate with NF-kB-p65 expression in neuroblastoma tumor samples, the role of NF-κB-p65 on CXCR4 expression was investigated *in vitro*. In response to an appropriate signal, the cytoplasmic inhibitor IκB-α is phosphorylated on serine and degraded, thus dissociating from the NF-κB (p65-p50) heterodimer. As a result, NF-κB heterodimer translocates from the cytosol to the nucleus and induces the expression of target genes containing NF-κB response elements (24). Previous studies (26,27) have shown that TNF-α treatment of various cell types stimulated NF-κB activation. To determine whether TNF-α activated NF-κB signaling in human neuroblastoma SH-SY5Y cells, SH-SY5Y neuroblastoma cells were cultured in the presence of 20 ng/ml TNF-α for the indicated time and subsequently the total cell lysates were collected and subjected to western blot analysis for NF-κB (p65), phosphorylated-IκB-α and IκB-α. Following exposure to TNF-α, SH-SY5Y cell lines showed nuclear translocation of NF-κB (p65) in the indicated time (Fig. 3A and B). Exposure to TNF-α increased IκB-α phosphorylation levels in the neuroblastoma SH-SY5Y cell lines, which was accompanied by a marked decrease in IκB-α protein expression (Fig. 3C and D).

**Figure 4:**
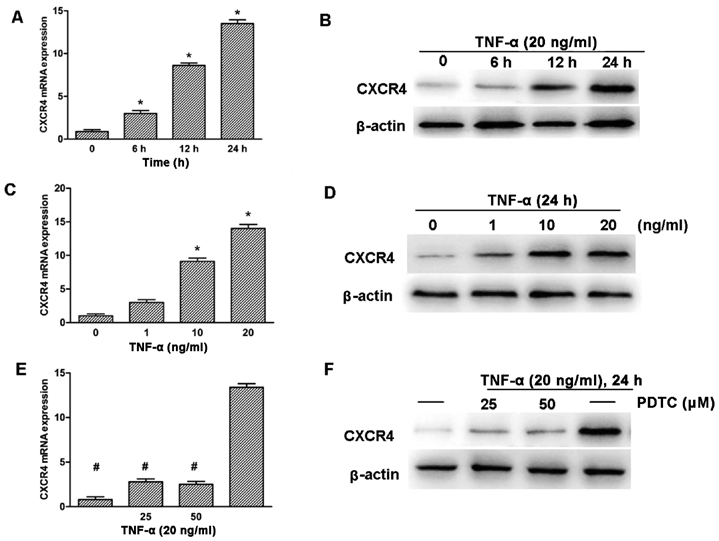
TNF-α promotes CXCR4 expression through NF-κB pathway activition in SH-SY5Y cells. (A and B) Cells were treated with TNF-α (20 ng/ml) for various time, as indicated. (C and D) Cells were treated with TNF-α for various concentrations, as indicated, for 24 h. (E and F) Cells were treated with NF-κB inhibitor PDTC (25 and 50 *μ*M) for 2 h and were subsequently treated with TNF-α (20 ng/ml) for 24 h. CXCR4 expression was analyzed by RT-qPCR (A, C and E) and western blot analysis (B, D and F). GAPDH and β-actin were used as the loading control. (^*^P<0.05 vs. untreated group, ^#^P<0.05 vs. TNF-α-treated group). TNF-α, tumor necrosis factor-α; CXCR4, CXC chemokine receptor-4; NF-κB, nuclear factor-κB; PDTC, pyrrolidinedithiocarbamic acid ammonium salt; RT-qPCR, reverse transcription-quantitative polymerase chain reaction.

To further explore the role of TNF-α in the upregulation of CXCR4 expression, SH-SY5Y neuroblastoma cells were treated with TNF-α for various time and concentrations. RT-qPCR and western blotting detection revealed that the expression of CXCR4 was upregulated significantly in a time- and dose-dependent manner (Fig. 4A–D). The CXCR4 promoter region has been shown to contain NF-κB response elements (16), thus, whether the NF-κB pathway played a role in the induction of CXCR4 in response to TNF-α was investigated. SH-SY5Y cells were pre-treated with PDTC, which is a potent antioxidant inhibitor of NF-κB (28). The cells were subsequently treated with 20 ng/ml TNF-α for 24 h. RT-qPCR and western blot analysis showed that PDTC clearly inhibited TNF-α-induced CXCR4 expression in SH-SY5Y cells (Fig. 4E and F).

### Upregulation of CXCR4 in SH-SY5Y cells is mediated by the NF-κB signaling pathway in the co-culture system

**Figure 5:**
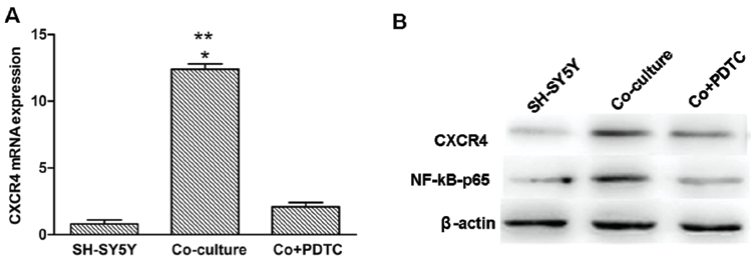
Macrophage-increased upregulation of *CXCR4* expression is dependent on the NF-κB signaling pathway in the co-culture system. (A and B) The SH-SY5Y cells expressed more CXCR4 following co-culture with THP-1 derived macrophages cells. The upregulation of CXCR4 expression was inhibited following treatment with PDTC. CXCR4 expression was measured by RT-qPCR and western blotting. NF-κB-p65 protein expression was measured by western blotting. GAPDH and β-actin were used as the loading control. (^*^P<0.05 vs. SH-SY5Y group. ^**^P<0.05 vs. CO+PDTC group). CXCR4, CXC chemokine receptor-4; NF-κB, nuclear factor-κB; PDTC, pyrrolidinedithiocarbamic acid ammonium salt; RT-qPCR, reverse transcription-quantitative polymerase chain reaction; CO, co-culture group.

In the present study, PDTC, a specific inhibitor of the NF-κB pathway was added to the macrophages/SH-SY5Y co-culture system for 24 h at a concentration of 50 *μ*M. This treatment resulted in a significant reduction in *CXCR4* mRNA and protein levels in the co-cultured SH-SY5Y cells when compared to the SH-SY5Y cells, either in the positive or negative control. NF-κB P65 protein was significantly decreased in the SH-SY5Y cells in the presence of PDTC (Fig. 5A and B).

### NF-κB mediates the migration towards SDF-1α in neuroblastoma cells

**Figure 6:**
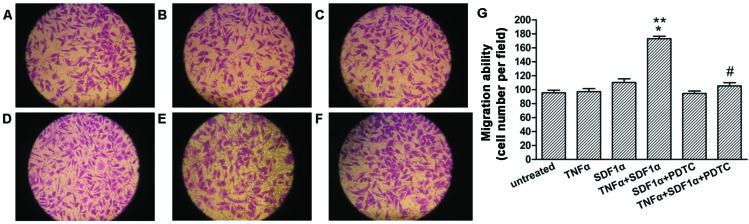
NF-κB mediates migration towards SDF-1α in the SH-SY5Y cells. SH-SY5Y cells pretreated or untreated with TNF-α (20 ng/ml) for 24 h were seeded in the migration chambers in the presence or absence of PDTC (50 *μ*M) and placed into wells in the presence or absence of SDF-1α (100 ng/ml). The cells were allowed to migrate for 24 h. Representative figures indicate the average number of migrated cells per field. (A) Untreated control. (B) TNF-α pretreated group. (C) Untreated control, SDF-1α-exposed group. (D) TNF-α pretreated, SDF-1α-exposed group. (E) PDTC pretreated group, SDF-1α-exposed group. (F) TNF-α, PDTC pretreated, SDF-1α-exposed group. (G) Migration ability for the different treatments. Results are representative of five independent experiments. ^*^P<0.05 vs. TNF-α pretreated group; ^**^P<0.05 vs. untreated, SDF-1α exposed group; ^#^P<0.05 vs. TNF-α pretreated, SDF-1α exposed group. NF-κB, nuclear factor-κB; TNF-α, tumor necrosis factor-α; SDF-1α, stromal cell-derived factor-1α; PDTC, pyrrolidinedithiocarbamic acid ammonium salt.

To evaluate the expression of NF-kB in regulating the migration of neuroblastoma cells towards SDF-1α, the transwell migration assay was performed. As shown in Fig. 6, TNF-α pre-treated cells showed a significant increase in migration towards SDF-1α as compared to cells exposed to SDF-1α alone or TNF-α pre-treated without SDF-1α in the lower well (Fig. 6B–D, P<0.05). Following knockdown of NF-κB expression with PDTC, the migration of the TNF-α pre-treated cells towards SDF-1α was significantly decreased (Fig. 6D and F, P<0.05).

## Discussion

Neuroblastoma is the most common malignant tumour in infancy, its high degree of malignancy and early metastasis are critical factors that affect the cure rate of neuroblastoma patients. However, the molecular and cellular mechanisms regulating neuroblastoma metastatic spread remain largely elusive.

Evidence indicating that inflammatory mediators affect genetic stability and cause persistent epigenetic alterations indicates that inflammatory components of the tumor microenvironment impact on the fundamental mechanisms responsible for the generation of metastatic variants. Inflammatory cytokines produced by the tumor or inflammatory cells in the tumor microenvironment promote tumor progression through the induction of genes dependent on NF-κB signaling pathway (29–31).

The role of the NF-κB signaling system in connecting inflammation and cancer is currently well accepted (32); furthermore, NF-κB is increasingly recognized as a crucial element in numerous steps of cancer initiation and progression. Elevated NF-κB activity is observed in various types of cancer, including neuroblastoma (33). The activation of NF-κB induces the expression of various molecules, including cyclooxygenase-2, matrix metallopeptidase-9 and adhesion molecules, such as intracellular adhesion molecule 1, vascular cell adhesion molecule 1 and endothelial-leukocyte adhesion molecule 1, all of which have been linked with cancer cell invasion and metastasis (34). The inhibition of NF-κB activity is believed to suppress neuroblastoma cell migration and invasion. TNF-α has been shown to induce NF-κB activation (35). The NF-κB complex is normally confined to the cytosol through its interaction with the IκB protein; upon stimulation, IκB is degraded and NF-κB is activated. In the present study, IκB-α phosphorylation and NF-κB nuclear translocation were observed in TNF-α-stimulated SH-SY5Y cells. These results are in agreement with a previous study, which revealed that TNF-α operates via activation of NF-κB pathways (36).

The CXCR4 chemokine receptor, which has been closely linked with cancer cell growth, invasion, angiogenesis and metastasis, has been found to be overexpressed in various types of tumor, including breast cancer, ovarian cancer, glioma, pancreatic cancer, prostate cancer, acute myeloid leukemia as compared to normal cells, which show little or no CXCR4 expression (37–39). A previous study indicated that a CXCR4/SDF-1α axis may be involved in attracting neuroblastoma cells to bone marrow, which was one of the favorable sites of metastasis formation by neuroblastoma (24); however, the mechanism responsible for its upregulation has not been completely elucidated. Although what leads to the overexpression of CXCR4 in cancer cells remains unclear, studies point to genetic and microenvironmental factors (35). Hypoxia in the tumor microenvironment (40) and NF-κB (16) have been indicated in *CXCR4* overexpression. In the present study, there was a significant positive correlation between the expression status of NF-κB-p65 and that of CXCR4 in neuroblastoma tissues. TNF-α was also shown to induce CXCR4 expression in neuroblastoma cells in a time- and dose-dependent manner. In addition, blocking the NF-κB pathway with PDTC suppressed TNF-α-induced CXCR4 expression. There was another clear upregulation of CXCR4 expression in SH-SY5Y cells following co-culture with macrophages, an alternative source of TNF-α in the neuroblastoma microenvironment. Notably, this upregulation was inhibited by the NF-κB inhibitor, PDTC.

Overexpression of *CXCR4*, whose involvement in various human tumors is well known, was frequently observed in neuroblastoma tissues to increase neuroblastoma metastasis. In the present study, there was a marked increase in migration towards SDF-1α in TNF-α pre-treated SH-SY5Y cells and the treatment with a NF-κB inhibitor, PDTC, resulted in a significant suppression of SH-SY5Y cell migration towards SDF-1α.

Taken together, these findings led to the conclusion that TNF-α partially functions through the NF-κB signaling pathway to upregulate *CXCR4* expression to foster neuroblastoma metastasis. Inflammatory factors in the tumor microenvironment activated NF-κB; constitutive NF-κB activation further upregulates major inflammatory factors, such as TNF-α, interleukin (IL)-6, IL-1 and IL-8, which are potent activators for NF-κB. Thus, it is believed that NF-κB and inflammation constitute a positive feedback loop to promote tumor cell survival and progression (41). However, the possibility of other transcription factors, in addition to NF-κB, contributing to the TNF-α-mediated upregulation of *CXCR4* should be considered. For instance, hepatocyte growth factor and hypoxia inducible factor-1 are able to activate the transcription of *CXCR4* (42).

Of note in the present study, the immunohistochemical analysis revealed significantly higher expression of NF-κB and CXCR4 in neuroblastoma tissues when compared to ganglioneuroma tissues, which further supports the increasing data that NF-κB and CXCR4 are abnormally expressed in neuroblastoma cells. Furthermore, there were significant correlations between the high level of NF-κB-p65, CXCR4 expression, clinical metastasis and INSS stages, which indicated the utility of NF-κB and CXCR4 as predictive biomarkers and therapeutic targets in neuroblastoma.

In conclusion, the inflammatory factor, TNF-α, promoted human SH-SY5Y cell metastasis through activation of NF-κB and upregulation of *CXCR4* expression. Inhibition of the TNF-α-activated NF-κB pathway suppressed cell migration in the SH-SY5Y cells.

The present data suggests that the TNF-α-activated NF-κB/CXCR4/SDF-1α pathway may be a potential regulator of neuroblastoma cell metastasis. Targeting inflammatory cytokines or NF-κB signaling pathways, and ultimately CXCR4, may be a therapeutic strategy in neuroblastoma.

## Figures and Tables

**Table I:** NF-κB-p65 and CXCR4 expression in neuroblastoma and their association with the clinicopathological characteristics.

Characteristics	Total cases, n	NF-κB-p65 expression			CXCR4 expression		
		Low (n=32)	High (n=48)	P-value	Low (n=23)	High (n=57)	P-value
Age, years							
<5	31	15	16	0.249	8	23	0.800
≥5	49	17	32		15	34	
Gender							
Male	48	17	31	0.356	11	37	0.209
Female	32	15	17		12	20	
Tumor size, cm							
<3	41	23	28	0.244	10	31	0.461
≥3	39	9	20		13	26	
INSS stages							
I, IIa, IVs	21	13	8	0.021^a^	12	9	0.0016^a^
IIb, III, IV	59	19	40		11	48	
Metastasis							
Absence	18	12	6	0.013^a^	11	7	0.002^a^
Presence	62	20	42		12	50	

a P<0.05. NF-κB, nuclear factor-κB; CXCR4, CXC chemokine receptor-4; INSS, International Neuroblastoma Staging System.

**Table II:** Expression of NF-κB-p65 and CXCR4 in neuroblastoma compared to ganglioneuroma tissues.

Variables	Total case, n	NF-κB-p65 expression			CXCR4 expression		
		Low, n	High, n	P-value	Low, n	High, n	P-value
Neuroblastoma	80	21	59	0.0001	24	56	0.0008
Ganglioneuroma	15	13	2	12	3		

NF-κB, nuclear factor-κB; CXCR4, CXC chemokine receptor-4.

**Table III:** Correlation of NF-κB-p65 and CXCR4 expression in neuroblastoma patient samples (P=0.0052).

NF-κB-p65	CXCR4 positive, n	CXCR4 negative, n	Total, n
Positive	21	18	39
Negative	9	32	41
Total	30	50	80

NF-κB, nuclear factor-κB; CXCR4, CXC chemokine receptor-4.
